# Paraneoplastic neurological syndromes associated with ovarian tumors

**DOI:** 10.1007/s00432-014-1745-9

**Published:** 2014-06-26

**Authors:** Mikolaj Piotr Zaborowski, Marek Spaczynski, Ewa Nowak-Markwitz, Slawomir Michalak

**Affiliations:** 1 Division of Gynecologic Oncology, Department of Gynecology, Obstetrics and Gynecologic Oncology, Poznan University of Medical Sciences, Polna str. 33, 60-535 Poznan, Poland; 2 Present Address: Department of Neurology, Massachusetts General Hospital, Harvard Medical School, Boston, MA 02129 USA; 3 Department of Neurochemistry and Neuropathology, Poznan University of Medical Sciences, Przybyszewskiego str. 49, 60-355 Poznan, Poland; 4 Neuroimmunological Unit, Polish Academy of Sciences, Przybyszewskiego str. 49, 60-355 Poznan, Poland

**Keywords:** Ovarian cancer, Ovarian teratoma, Paraneoplastic cerebellar degeneration, Anti-NMDAR encephalitis

## Abstract

**Introduction:**

Paraneoplastic neurological syndromes (PNS) are neurologic deficits triggered by an underlying remote tumor. PNS can antedate clinical manifestation of ovarian malignancy and enable its diagnosis at an early stage. Interestingly, neoplasms associated with PNS are less advanced and metastasize less commonly than those without PNS. This suggests that PNS may be associated with a naturally occurring antitumor response.

**Methods:**

We review the literature on the diagnosis, pathogenesis and management of PNS associated with ovarian tumors: paraneoplastic cerebellar degeneration (PCD) and anti-*N*-methyl-d-aspartate receptor (anti-NMDAR) encephalitis. An approach to the diagnostic workup of underlying tumors is discussed.

**Results:**

PCD can precede the manifestation of ovarian carcinoma. Anti-NMDAR encephalitis in young women appears often as a result of ovarian teratoma. Since ovarian tumors and nervous tissue share common antigens (e.g., cdr2, NMDAR), autoimmune etiology is a probable mechanism of these neurologic disorders. The concept of cross-presentation, however, seems insufficient to explain entirely the emergence of PNS. Early resection of ovarian tumors is a significant part of PNS management and improves the outcome.

**Conclusions:**

The diagnosis of PNS potentially associated with ovarian tumor indicates a need for a thorough diagnostic procedure in search of the neoplasm. In some patients, explorative laparoscopy/laparotomy can be considered.

## Key points

- PNS precede clinical manifestation of ovarian tumors and enable their diagnosis at an early stage.
- Paraneoplastic cerebellar degeneration (PCD) can coexist with ovarian carcinoma.
- Anti-NMDAR antibodies detected in patient affected with encephalitis are highly suggestive of ovarian teratoma.
- Ovarian tumors and nervous tissue share common antigens in PNS (e.g., cdr2, NMDA receptor)—the concept of cross-presentation, however, may not be sufficient to explain an emergence of PNS.
- Cell-mediated immune response plays a role in the pathogenesis of PNS.
- Antibody-mediated immune response is a major mechanism of anti-NMDAR encephalitis and other NSAS.
- In patients with PNS associated potentially with ovarian tumors, transvaginal ultrasound examination and pelvic CT scan are indicated—if negative, PET imaging is required.
- If imaging studies remain normal, explorative laparoscopy/laparotomy should be considered.
- An early resection of ovarian tumors is a significant part of PNS management and improves the outcome.

## Definition

Paraneoplastic neurological syndromes (PNS) are defined as the pathologic involvement of the nervous system in the course of malignancy. This entity does not include tumor infiltration, compression or metastasis of the nervous system (Graus et al. 2004). PNS often precede clinical manifestation of a tumor and enable diagnosis at an early stage. Interestingly, neoplasms that appear in patients with PNS are limited in size and metastasize less commonly than those without PNS (Hetzel et al. 1990; Albert and Darnell 2004). These observations suggest that PNS provide an example of a naturally occurring antitumor response. Ovarian tumors account for about 10 % of malignancies associated with PNS (Giometto et al. 2010). Paraneoplastic cerebellar degeneration (PCD), the most common paraneoplastic neurological syndrome, may coexist with ovarian carcinoma (Giometto et al. 2010). Recently, a group of neurologic disorders, neuronal surface antibodies syndromes (NSAS), has been defined (Zuliani et al. 2012). They usually affect the central nervous system in the form of encephalitis that manifests mainly as behavioral disorder and seizures. However, they are rarely related to malignancy. One of NSAS, however, anti-NMDAR encephalitis, appears typically in young women with a benign tumor, ovarian teratoma.

The diagnosis of PNS is based on criteria defined in Graus et al. (2004). This takes into account neurological symptoms, presence of a tumor and antibodies associated with this condition, referred to as onconeural antibodies (Graus et al. 2004). The diagnosis of PNS is a complex process that always requires exclusion of primary etiology of neurologic symptoms, including among others—vascular disorders, infection, nervous tissue tumors or hereditary syndromes. The classification of PNS distinguishes “classical syndromes” and “well-characterized onconeural antibodies” that are the most specific for PNS (Tables 1, 2). The term “definite PNS” in contrast to “possible PNS” indicates higher probability of the paraneoplastic nature of a disorder (Table 3). This differentiation is important since definite PNS requires a more intense search for underlying tumor. Neuronal surface antibodies syndromes typically respond well to immunotherapy. Hence, once the diagnosis of anti-NMDAR encephalitis is established, it is classified as “definite NSAS” if it is responsive to immunomodulatory agents such as steroid and intravenous immunoglobulin and as “probable NSAS” if not responsive (Zuliani et al. 2012).

**Table 1:** Classical paraneoplastic syndromes

Classical paraneoplastic neurological syndromes
Encephalomyelitis
Limbic encephalitis
**Subacute cerebellar degeneration**
Opsoclonus–myoclonus
**Subacute sensory neuronopathy**
Chronic gastrointestinal pseudo-obstruction
Lambert–Eaton myasthenic syndrome
Dermatomyositis

PNS the most commonly associated with ovarian tumors are in bold (Graus et al. 2004)

**Table 2:** Onconeural and neuronal surface antibodies

Onconeural and neuronal surface antibodies		
Well characterized	Partly characterized	Neuronal surface antibodies
Anti-Hu **Anti-Yo** **Anti-Ri** Anti-CV2 Anti-Ma **Anti-amphiphysin**	Anti-Tr Anti-Zic4 Anti-mGluR1 ANNA3 PCA2	Anti-VGKC complex antigens (LGI1 or CASPR2) **Anti-NMDAR** Anti-AMPAR Anti-GABABR Anti-GlyR Anti-VGCC-Ab Anti-mGluR1 Anti-mGluR5

Antibodies detected commonly in PNS associated with ovarian tumors are in bold (Graus et al. 2004; Titulaer et al. 2011; Zuliani et al. 2012)

**Table 3:** Definite and possible PNS according to diagnostic criteria as published by Graus et al. (2004)

Definite PNS	Possible PNS
*Definite and possible diagnosis of PNS*	
1. A classical syndrome and cancer that develop within 5 years of the diagnosis of the neurological disorder	1. A classical syndrome, no onconeural antibodies, no cancer is diagnosed, but at high risk for having an underlying tumor
2. A non-classical syndrome that resolves or significantly improves after cancer treatment without concomitant immunotherapy provided that the syndrome is not susceptible to spontaneous remission	2. A neurological syndrome (classical or not) with partially characterized onconeural antibodies and no cancer
3. A non-classical syndrome with onconeural antibodies (well characterized or not) and cancer that develops within 5 years of the diagnosis of the neurological disorder	3. A non-classical syndrome, no onconeural antibodies and cancer present within 2 years of diagnosis
4.** A neurological syndrome (classical or not) with well-characterized onconeural antibodies (anti-Hu, Yo, CV2, Ri, Ma2 or amphiphysin) and no cancer**	

Neurological syndrome with well-characterized antibodies (in bold) is a clinical setting that requires intense search for underlying malignancy and often enables its detection at an early stage. Reproduced from ‘Recommended diagnostic criteria for paraneoplastic neurological syndromes’ (Graus et al. 2004) with permission from BMJ Publishing Group Ltd.

## Incidence

Paraneoplastic syndromes are rare in cancer patients. In the group of 1,465 unselected patients with various tumors, the history and physical examination revealed neurologic abnormalities in 96 individuals (Croft and Wilkinson 1965). In 55 women with ovarian cancer, neurologic disorder was diagnosed in nine patients (Croft and Wilkinson 1965). These data should, however, be interpreted with caution as since the time this study was performed the diagnostic criteria of PNS have evolved markedly. It is estimated that paraneoplastic neurological syndromes appear in about 1 % of all malignancies (Rees 2004). The diagnosis, however, becomes more probable in particular contexts—approximately half of patients with non-familial cerebellar syndrome of subacute onset suffer from some malignancy, primarily ovarian or lung cancer. The criteria for NSAS diagnosis were clearly defined in 2012 (Zuliani et al. 2012). Antibodies in anti-NMDAR encephalitis typically associated with ovarian teratoma were described for the first time in 2005 (Vitaliani et al. 2005). In over 3 years, more than 400 cases of NSAS have been reported (Dalmau et al. 2011). This entity can affect adolescents and children, as well as adults. It is worth mentioning that three cases of anti-NMDAR encephalitis occurred during pregnancy complicated with ovarian teratoma (Kumar et al. 2010). Though PNS are still considered rare, their estimates of incidence increase with the improvement in diagnostic tools.

## Diagnosis

Paraneoplastic neurological syndromes may be diagnosed prior to tumor detection. It is worth stressing that a combination of classical PNS and the detection of well-characterized antibodies are known to be involved in PNS, even without established diagnosis of malignancy is also referred to as definite paraneoplastic disorder (Table 3). In such cases, there is an obligation to screen for underlying malignancy (Titulaer et al. 2011). In a group of 55 patients with PCD the neurological symptoms antedated the diagnosis of a neoplasm in 34 individuals (Peterson et al. 1992). In 18 out of 19 women with gynecologic cancer, primarily ovarian carcinoma, neurologic pathology preceded the detection of malignancy (Hetzel et al. 1990). The time between the onset of paraneoplastic syndrome and diagnosis of a tumor varies from a few weeks to months or even years (Hetzel et al. 1990; Rojas et al. 2000). Thus, PNS symptoms enable detection of a neoplasm at a very early stage. Rarely, paraneoplastic neurological syndrome may emerge after completed treatment of ovarian cancer (Forgy et al. 2001; Goldstein et al. 2009; Russo et al. 2013). Unfortunately, the paraneoplastic reaction is sometimes so intense that it itself worsens significantly the patient’s clinical outcome and leads to disability (Cao et al. 1999). Paraneoplastic cerebellar syndromes are highly debilitating and often irreversible (Mason et al. 1997). By contrast, anti-NMDAR encephalitis that affects women with ovarian teratoma responds well to tumor resection and subsequent immunotherapy which may lead to almost complete recovery.

### Clinical presentation

The onset of signs and symptoms of paraneoplastic syndrome is usually subacute and develops within a few weeks. PCD manifests clinically as cerebellar syndrome. The patient suffers from incoordination of movements (ataxia), balance and gait disturbances, speech disorder (dysarthria) and altered ocular movements (nystagmus, often in a downbeat form) (Peterson et al. 1992; Dalmau and Rosenfeld 2008). Paraneoplastic neurological syndromes associated with ovarian tumors may also appear as a peripheral polyneuropathy with diffuse paresthesia and anesthesia (Cao et al. 1999). Young women or children with ovarian teratoma can be affected with encephalitis that manifests as psychosis, memory loss and behavior disorder. It subsequently develops into seizures, dyskinesias and autonomic instability (Florance et al. 2009; Tanyi et al. 2012; Zuliani et al. 2012). Neurologic pathology is often highly debilitating and renders many patients either wheelchair bound or bedridden (Frings et al. 2005).

### Antibodies

Laboratory analyzes may reveal the presence of onconeuronal antibodies. They are detected by means of an indirect immunofluorescence and confirmatory Western or line blot (Monstad et al. 2008; Stefens-Stawna et al. 2013). The most common type of antibody found in PCD is anti-cdr2 (cerebellar degeneration protein-2 antibody) (also known as anti-Yo) (Anderson et al. 1988; Peterson et al. 1992). Anti-Yo antibody seropositive status suggests ovarian cancer, breast cancer or other gynecologic malignancies (Peterson et al. 1992). Infrequently, it can be related to small cell lung cancer (Greenlee and Lipton 1986). Recently, new antibodies, termed anti-cdr2-like (cdr2L), have been described (Eichler et al. 2013). They are reactive against an antigen similar to the cdr2 protein, which instead of being cytoplasmic is within the cell membrane. The coexistence of antibodies to cdr2 and cdr2L is strongly suggestive of definite PNS. Surprisingly, PCD has been diagnosed only in patients with both antibodies. Paraneoplastic cerebellar syndrome may also be associated with anti-Hu antibodies which raises suspicion of small cell lung cancer. The detection of anti-Ri, in turn, suggests an underlying breast cancer. Clinician should also take into consideration the diagnosis of lymphoma, especially if anti-Tr or anti-mGluR1 antibodies are identified (Shams’ili et al. 2003). Interestingly, PCD related to anti-Yo antibodies is associated with a worse survival due to cancer as compared to other antibodies (Giometto et al. 2010). In a group of neuronal surface antibodies syndromes, anti-NMDAR antibodies are strongly indicative of ovarian teratoma (Florance et al. 2009; Zuliani et al. 2012). A substantial number of patients, however, manifest paraneoplastic syndromes unrelated to detection of any of the above antibodies (Giometto et al. 2010). In addition, onconeuronal antibodies appear in some patients with ovarian tumors who do not suffer from any neurological symptoms. In a group of 181 women with ovarian cancer without paraneoplastic syndrome, there were four who had anti-Yo and seven who had anti-Ri onconeuronal antibodies (Drlicek et al. 1997). The detection of anti-cdr2 antibodies in ovarian cancer can be improved by in vitro transcription–translation (ITT) of radiolabelled cdr2 protein and immunoprecipitation assay (Monstad et al. 2006; Eichler et al. 2013). Anti-NMDAR antibodies were detected in six out 21 women with teratoma (Michalak et al. 2010). In another study, however, anti-NMDAR antibodies were not found among 20 neurologically asymptomatic patients with ovarian teratoma (Mangler et al. 2013). In general, ovarian tumors have high potential to mount an immune response, since anti-ovarian autoantibodies are commonly found in patients affected with these neoplasms (Szubert et al. 2012). The detection of antibodies is a major reason to raise the suspicion of paraneoplastic syndrome in a patient who manifests neurologic symptoms. The type of antibody indicates the most probable tumor location, including ovarian cancer and ovarian teratoma.

### CSF analysis

An analysis of cerebrospinal fluid (CSF) may display pleocytosis (with a high fraction of lymphocytes), elevated protein or oligoclonal bands (Frings et al. 2005; Rubello et al. 2005; Tanyi et al. 2012). Inflammatory changes and anti-NMDAR antibodies in CSF are frequently present in anti-NMDAR encephalitis (Vitaliani et al. 2005; Dalmau et al. 2011). In some patients with PCD, however, no abnormalities have been detected in the CSF (Peterson et al. 1992). Though the analysis of CSF often reveals disturbances in PNS, it is more informative in anti-NMDAR encephalitis than in PCD.

### Imaging studies

Magnetic resonance imaging (MRI) in PCD can reveal cerebellar atrophy (Frings et al. 2005). This is consistent with pathological *postmortem* examination, which demonstrates significant loss of Purkinje cells and sometimes coexistent infiltrates within cerebellum. In some PCD patients, however, both MRI and CT scan can appear normal (Negishi et al. 2013). The fluorodeoxyglucose positron-emission tomography (FDG-PET), in turn, imaging most commonly reveals cerebellar hypometabolism in PCD (Basu and Alavi 2008). Rarely, increased FDG uptake, consistent with hypermetabolism, is found in the cerebellar region (Choi et al. 2006). Imaging studies are useful mainly for differential diagnosis of cerebellar disorders. They help to exclude other conditions related to neoplasm that may give rise to neurologic symptoms, such as metastases, infiltration or vascular complications. MRI in anti-NMDR encephalitis in 50 % of patients shows hyperintensity in various brain regions, including hippocampus, corpus callosum, temporal and frontal lobes, while in other patients the brain image appears normal (Dalmau et al. 2011; Tanyi et al. 2012). Though imaging studies are not key to establish the diagnosis of PNS so far, they are indispensable in excluding other conditions responsible for neurologic symptoms.

### Electroencephalogram (EEG)

The EEG is pathological in the majority of patients with anti-NMDAR encephalitis. Abnormally slow and disorganized activity is usually not associated with anomalous movements and is unresponsive to antiepileptic therapy (Dalmau et al. 2008, 2011). It is consistent with a diffuse affection of brain tissue associated with encephalitis.

## Ovarian tumors

PNS can provide an opportunity to investigate naturally occurring antitumor immunity (Albert and Darnell 2004). In Hu syndrome associated with small cell lung cancer, the beneficial effect on survival of early detection due to PNS symptoms is well proven and several cases of tumor regression have been reported (Darnell and DeAngelis 1993; Graus et al. 1997). It is a question of debate, however, whether such a phenomenon applies to all tumors, including ovarian cancer. Specific cytotoxic T lymphocytes active against cdr2 antigen expressed by ovarian cancer and Purkinje cells were identified in peripheral blood in patients with PCD (Albert et al. 1998). In some patients with ovarian cancer coexisting with PCD, tumors are limited in size and only discovered by microscopic examination (Peterson et al. 1992) and have fewer secondary foci in comparison with patients without PCD (Hetzel et al. 1990). By histopathological analysis, these tumors are characterized as lesion with intense lymphocyte infiltration characteristic of an immune response (Peterson et al. 1992; Cao et al. 1999). On the other hand, in another study, metastatic disease was discovered in 15 out of 18 patients with ovarian cancer and PCD, with a median survival of 22 months comparable to patients with this form of cancer without PCD (Rojas et al. 2000). However, it can be hard to estimate an effect of antitumor response on overall survival as nearly half of the patients with PNS die from neurologic pathology (Rojas et al. 2000). Currently, in PCD associated with ovarian cancer, there is no study that explicitly corroborates an effective antitumor response in terms of prognosis. Further, the presence of anti-Yo antibodies in neurologically normal patients with ovarian cancer had no influence on survival (Drlicek et al. 1997). Hence, the antitumor reaction is probably effective at the very beginning of tumor development, so it less advanced at the time of diagnosis. For unknown reason, this response fails to eliminate cancer cells and the tumor progresses in a natural aggressive way. The nature of antitumor reaction in ovarian cancer coexistent with PCD requires more research.

Ovarian tumors associated with anti-NMDAR encephalitis include mainly teratomas (Dalmau et al. 2007, 2011). Among 91 women with anti-NMDAR encephalitis, 49 had ovarian teratoma, including 17 immature tumors and eight bilateral ovarian lesions (Dalmau et al. 2008). Cases of sex cord–stromal tumors coexistent with anti-NMDAR encephalitis were also reported (Tanyi et al. 2012). Pathological studies revealed the presence of nervous tissue with the expression of NR2 subunit of NMDA receptor within all teratomas (Dalmau et al. 2007, 2008). Significant inflammatory infiltrates were also demonstrated in tumors associated with anti-NMDAR encephalitis (Tüzün et al. 2009). The strong link between anti-NMDAR encephalitis and ovarian teratomas incidence can be well explained by the cross-presentation of the same antigen.

## Pathogenesis

**Fig. 1:**
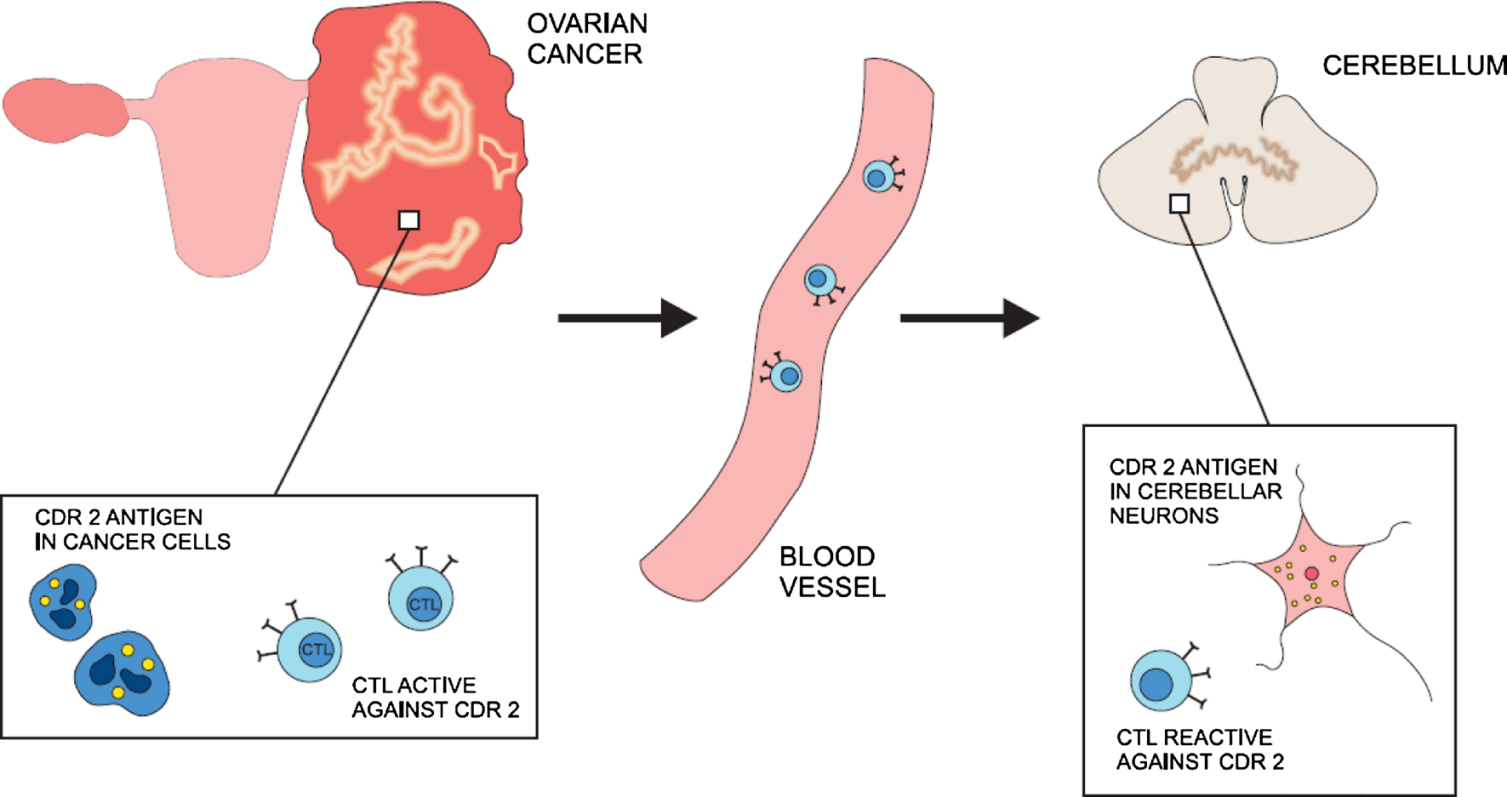
Ovarian cancer cell expresses cdr 2 antigen that triggers immune response against malignancy. The same cdr2 antigen is a intracellular protein in Purkinje cell in cerebellum. As a result, cytotoxic T lymphocytes (CTL) cross-react against nervous tissue. This mechanism represents the prevailing view on the pathogenesis of paraneoplastic cerebellar degeneration (PCD) related to ovarian cancer. The hallmarks of immune reaction, however, are not detected in all patients

Autoimmune processes undoubtedly take part in pathogenesis of PNS (Darnell and Posner 2003). The major hypothesis about the origins of PNS states that tumors express antigens that are normally present almost exclusively in nervous tissue (Fig. 1). The presentation of neuronal antigens by a neoplasm then mounts an intense immune response against the tumor which cross-reacts with the nervous tissue. To study this hypothesis, investigators have searched for onconeuronal antigens common to both nervous tissue and ovarian tumors. Sera of patients affected with PCD were incubated with ovarian cancer tissue from individuals without neurologic pathology (Darnell et al. 2000). In 13 out of 20 tissues, an antigen cdr2 (also called Yo protein) was detected in both cerebellar neurons and ovarian tumors. This cdr2 antigen was also expressed in 2 out of 9 breast cancer specimens as well. In one study, the protein was found in a normal ovary (Totland et al. 2011). It appears that many ovarian cancers express this protein, irrespective of the presence of anti-Yo antibodies or manifestation of PCD in patients (Darnell et al. 2000; Totland et al. 2011). The same relationship was observed in paraneoplastic neurological syndromes that accompany other malignancies, for example, with all small cell lung cancers possessing the Hu antigen and only some patients developing paraneoplastic Hu syndrome (Manley et al. 1995). Thus, the presence of onconeuronal antigens and antibodies against them does not completely correlate with the origin of paraneoplastic reaction.

A detection of onconeuronal antibodies is important for the syndrome diagnosis, even though it is not compulsory according to diagnostic criteria (Graus et al. 2004). The importance of this humoral response in the pathomechanism of the disease, however, is unclear. The passive transfer of antibodies to onconeuronal antigens did not lead to neurologic pathology in mouse model (Tanaka et al. 1995). Further, immunization of mice with human cdr2 antigen induced only antibody production in the absence of nervous tissue damage (Tanaka et al. 1994, 1995). It appears that lymphocytes taken from patients’ blood samples were also active against the same antigens as antibodies used in diagnosis (Albert et al. 1998; Tanaka et al. 1998; Albert et al. 2000; Zaborowski and Michalak 2013). Specific cytotoxic T lymphocytes have been found both in acute and chronic phase of PCD. In PCD associated with the anti-Yo antibody, a high frequency of HLA A24 has been demonstrated (Tanaka and Tanaka 1996). This observation suggests that patients in this group are more susceptible to autoimmune disorder, which is consistent with the involvement of cytotoxic T lymphocytes. It seems, on the other hand, that cell-mediated reaction is not always involved in neuronal damage in the course of PNS, as pathology studies do not always reveal infiltrates that accompany significant neuronal loss (Peterson et al. 1992) and a number of patients with PCD lack antibodies to onconeuronal antigens (Anderson et al. 1988). In rat models of neoplastic disease, cerebellar degeneration with Purkinje cell loss has been described without any detectable onconeuronal antibodies (Michalak 2008). The recent identification of anti-cdr2L antibodies may shed new light on the mechanism involved in PCD emergence (Eichler et al. 2013), and other, as yet unidentified, onconeural antigens may still be discovered. In contrast to the cdr2 protein that is cytoplasmic, cdr2L antigen is present at the cell membrane which may make it more accessible for antibody-mediated cytotoxicity. Moreover, when both present, anti-cdr2 and anti-cdr2L antibodies had much higher avidity than when detected alone. It was consistent with the fact that only patients with both antibody types developed definite PNS in contrast to individuals with either anti-cdr2 or anti-cdr2L alone. Cdr2L antigen is expressed both in Purkinje cells in cerebellum and in ovarian tumor tissue. The role of anti-cdr2L in the mechanism of PCD certainly requires further exploration. Taken together, the immune reaction, especially cell-mediated response, is an important player in the origins of PCD. Contradictory results of research in animal models, however, suggest that other factors potentially deleterious to nervous tissue may also be involved.

Cdr2 is involved in the inhibition of c-myc oncoprotein that is widely expressed in many tumors, including ovarian cancer (Okano et al. 1999). Cdr2 sequesters c-myc and prevents it from inducing transcription. Cdr2 expression presumably represses cell proliferation through this mechanism, functioning as a tumor suppressor. Elevated expression of cdr2 in a tumor tissue may lead to immune responses that are also active against nervous tissue. Over-expression of cdr2 could explain why PCD appears in neoplasms that have a less aggressive course, since suppressive mechanisms mediated by cdr2 are constitutively more effective. Since in PNS, tumors have often intense infiltrates and the significance of cell-mediated immune response has been revealed, it is probable that immune responses eliminate cancer cells.

**Fig. 2:**
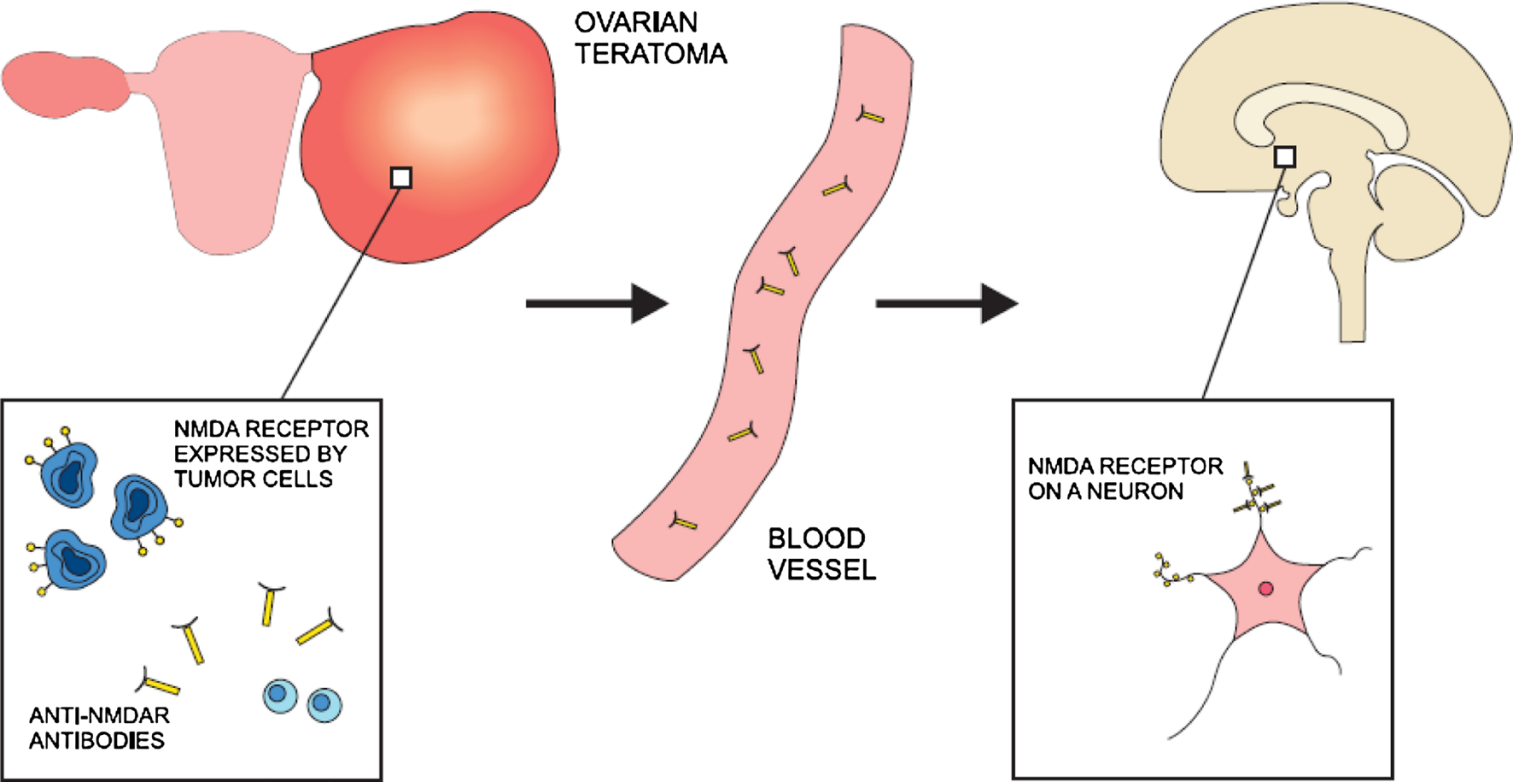
NMDA receptor (NMDAR) is expressed on the surface of ovarian teratoma cells. As a result of immune reaction, anti-NNMAR antibodies are produced. The same receptor is present at the dendrites of neurons in many region of central nervous system. Anti-NMDAR antibodies enter nervous tissue through the vasculature and react against the target protein. Consequently, neural signaling mediated by NMDA receptor is considerably disturbed leading to both psychiatric and neurologic symptoms

By contrast, the pathogenic role of antibodies seems well proven in anti-NMDAR encephalitis (Fig. 2). In vitro studies revealed that rat neurons incubated with patients’ sera containing anti-NMDAR antibodies had reduced numbers of NMDA receptors in postsynaptic dendrites. This phenomenon was reversible after treatment with control CSF (Dalmau et al. 2008). Moreover, in anti-NMDAR encephalitis, clinical improvement is associated with a decrease in NMDR antibody titers (Dalmau et al. 2008). Pathological studies have demonstrated numerous antibody deposits and scarce cell infiltrates in affected regions of brain (Tüzün et al. 2009). Notably, the NMDR antigens for these antibodies were identified mainly in hippocampus (Vitaliani et al. 2005). In contrast to intracellular onconeuronal antigens, the target protein was detected within the plasma membrane which makes it more accessible to antibodies. A favorable clinical response to plasma exchange also provides an argument in favor of antibody-mediated mechanism of disease. NMDAR antigen has been found in all ovarian teratomas excised in the course of anti-NMDAR encephalitis (Tüzün et al. 2009). These observations suggest that the cross-presentation of NMDAR antigen in neurons and ovarian tumor leads to the anti-NMDAR antibodies production, which induces encephalitis.

## Management

Diagnosis of PCD should always be associated with a search for a primary origin of malignancy. This nervous system pathology is most commonly seen in ovarian, breast and small cell lung cancers or lymphoma (Peterson et al. 1992). In some patients, an ovarian tumor can be visualized by transvaginal ultrasound or by pelvic CT scan (Negishi et al. 2013). The neoplastic disease, however, is frequently at a very early stage and consequently a conventional diagnostic procedure may appear inconclusive. A number of studies show that the use of FDG-PET imaging is more sensitive and justified in this clinical context (Frings et al. 2005). An ovarian tumor that was not visible in transvaginal ultrasound appeared evident on the integrated PET–CT scan (Marchand et al. 2007). Surgery confirmed the diagnosis of a papillar serous adenocarcinoma of an ovary at stage IIB. An annual follow-up of another patient with PCD uncovered hyperactivity of an axillary region 5 years after the onset of neurologic symptoms (Mathew et al. 2006). The biopsy was consistent with metastasis of breast cancer. In some patients, combined FDG-PET imaging is the only one that can reveal the location of the malignancy (Rubello et al. 2005).

It is rather the antigenic specificity than particular neurologic syndromes that is more indicative for tumor location (Titulaer et al. 2011). The most specific for diagnosis, however, is the combination of both. In patients with anti-Yo, anti-Ri or anti-amphiphysin antibodies ovarian cancer should be suspected. If anti-NMDAR antibody is detected, it suggests coexistence of teratoma, including its immature type. The metanalysis of numerous studies conducted by Titulaer et al. (2011) contains conclusions concerning management. In all these patients, transvaginal ultrasound is the investigation of choice. If it is negative, pelvic computed tomography or magnetic resonance imaging should be performed. In patients with antibodies that suggest ovarian cancer, an integrated FDG-PET/CT is indicated. It is important to perform it as soon as possible as combined PET/CT scan used in this context may significantly reduce the delay time to the surgery (Frings et al. 2005). If initial investigations do not detect any tumor, it is recommended to repeat them every 6 months for 4 years.

PNS develop as a result of the malignancy. Thus, it is not surprising that the neurological outcome in PCD may improve after tumor excision. Subsequent systemic immune therapy in the form of intravenous immunoglobulin, steroids and tacrolimus has also proven beneficial (Negishi et al. 2013). In young women affected with NMDR encephalitis associated with teratoma, surgical removal of the tumor in combination with plasma exchange, steroid and intravenous immunoglobulin may yield almost complete recovery (Tanyi et al. 2012). In some patients, a second-line immunotherapy (rituximab or cyclophosphamide) is necessary (Dalmau et al. 2011). It has been proven that early surgery combined with immunotherapy markedly improves outcome (Dalmau et al. 2008).

The question arises whether the diagnosis of paraneoplastic reaction commonly associated with ovarian tumor may itself constitute an indication for surgical management. Exhaustive laboratory and imaging investigations often remain inconclusive as to the presence and location of the tumor. Some reports suggest, however, that explorative surgery may reveal an occult neoplasm. In a group of 19 patients with both ovarian cancer and paraneoplastic cerebellar syndrome, laparotomy was performed (Hetzel et al. 1990). In seven women, there were no tumor symptoms and the laboratory as well as imaging investigation was normal prior to the surgery. The decision on operative management was taken exclusively based on the detection of onconeuronal antibodies. Surprisingly, all these patients had high-grade ovarian adenocarcinoma. Another remarkable observation made by the authors was either the lack of or limited volume of peritoneal invasion that are typical for advance ovarian cancer. In another study, laparotomy performed in some patients with PCD uncovered solely microscopic foci of ovarian cancer (Peterson et al. 1992). These findings are consistent with a report of *postmortem* examination of a patient who died due to very severe paraneoplastic reaction affecting the nervous system (BRAIN et al. 1951). It revealed two very small ovarian tumors the size of which did not exceed 2 cm in diameter. These phenomena suggest that paraneoplastic reaction corresponds to an antitumor response. The history of a patient with PCD and abnormal CA-125 levels at postmenopausal age has also been reported (Mason et al. 1997). Though imaging studies did not suggest ovarian pathology, both laparoscopic salpingo-oophorectomy and endometrial biopsy were performed. The pathological examination did not confirm malignancy; however, the CA-125 level decreased to normal limits after ovarian resection. Another report presented the history of a patient with PCD whose retroperitoneal lymph node was enlarged to the size of 2.2 cm and Ca125 was highly elevated. Though during laparotomy adnexa were normal macroscopically, pathological examination revealed the presence of a microscopic adenocarcinoma of the fallopian tube (Frings et al. 2005). In a 53-year-old patient with anti-NMDAR encephalitis whose neurologic and psychiatric condition was deteriorating, the decision of abdominal laparoscopy was made in spite of normal abdominal and pelvic CT scan. Though both adnexa were macroscopically normal, the pathologic examination revealed a sex cord tumor (Tanyi et al. 2012). In a group of adult women affected with anti-NMDAR encephalitis, 56 % had ovarian teratoma (Florance et al. 2009). It should be noted that this fraction was significantly lower in adolescents and children (31 %). Ovarian resection in patients with PNS without clear diagnosis of cancer can be considered effective in terms of both malignancy and neurologic outcome. This can have considerable value in neoplasm therapy, for often the surgery reveals very early stage disease. It is also suggested by some authors that an early management of malignancy may prevent a more severe neurologic deterioration that may remain irreversible and highly debilitating otherwise (Vedeler et al. 2006; Tanyi et al. 2012). It has been confirmed that antitumor treatment improves overall survival in these patients (Shams’ili et al. 2003). In anti-NMDAR encephalitis, an emergent surgery contributes substantially to the final outcome and may enable even almost complete recovery (Dalmau et al. 2007; Florance et al. 2009; Tanyi et al. 2012). Even if serial CT or ultrasound is not suggestive of teratoma, an explorative surgery may prove informative. In a woman affected with anti-NMDAR encephalitis, blind oophorectomy was performed revealing an underlying teratoma and the resection was associated with significant neurological improvement (Johnson et al. 2010). In contrast, an excision of ovaries in combination with immunotherapy led to clinical improvement in another patient, but surprisingly the pathological examination was normal (Parratt et al. 2009). Taken together, it seems reasonable to perform an explorative laparoscopy or laparotomy in patients with paraneoplastic cerebellar syndrome or anti-NMDAR encephalitis whose imaging studies appear normal. After precise exclusion of other neurological aetiologies and considering the patient’s age, the salpingo-oophorectomy of macroscopically normal adnexa may appear beneficial.

## Summary

The clinical manifestation of ovarian tumors can be preceded by neurological deficit, particularly in cases with ovarian cancer or teratoma. The detection of onconeural antibodies (e.g., anti-Yo) strongly indicates the presence of ovarian tumor in patients with neurological deficit. If the tumor is not identified, an obligation for systematic screening exists. Surgery and immunomodulatory treatment are considered as the most important management among such a group of patients.
